# Supplementary material for: The genome sequence of the Gypsy Moth, Lymantria dispar Linnaeus, 1758
Source: Wellcome Open Res. 2025 Jan 30;10:37. [Version 1] doi: 10.12688/wellcomeopenres.23626.1 (PMC12661151; doi:10.12688/wellcomeopenres.23626.1)
Supplement: Peer review history

# Reviewer report: Reviewer response for version 1

**DOI:** 10.21956/wellcomeopenres.26066.r139064

**Published:** 2026-03-06

**Authors:** Jason Charamis

**Affiliations:** 1 Foundation for Research and Technology - Hellas, Irákleion, Greece

This manuscript describes the genome sequencing and assembly of the gypsy moth *Lymantria dispar*. The work is technically sound, the assembly is chromosome-level and the backgrounds, methods and results are nicely described. I do not have any further comments or suggestions to make. I think that the paper should be accepted. 

 This "Data Note" presents a chromosome-level genome assembly for the Gypsy Moth (Lymantria dispar), a significant defoliator of broadleaved trees in the Northern Hemisphere. The assembly was generated using PacBio HiFi long-read sequencing and Hi-C data for scaffolding. The resulting genome is 966.90 Mb in length with a scaffold N50 of 35.2 Mb, while 99.94% of the sequence is assigned to 32 chromosomal pseudomolecules (including W and Z sex chromosomes). The assembly achieves a high quality value (QV) of 66.2 and a BUSCO completeness score of 98.9%, indicating an excellent assembly. The manuscript also details the annotation of 16,242 protein-coding genes via the Ensembl pipeline. The identification and assembly of both W and Z sex chromosomes is a notable technical achievement, as the heterogametic sex (female in Lepidoptera) often presents assembly challenges regarding sex chromosome differentiation.   Minor Comments 1. Ambiguity in Sample Preparation (Anatomy) The methodology states that "Tissue from the abdomen was homogenised" for DNA extraction. As noted by the existing peer reviewer, this description is insufficiently precise for a larval specimen. In lepidopteran larvae, the "abdomen" is dominated by the midgut, which contains plant material and a complex microbiome. While the authors mention an "Assembly Screen for Cobionts and Contaminants (ASCC) pipeline", it is unclear if the gut was dissected out to reduce non-host DNA load, or if the whole abdominal segment was used. Clarifying this is essential for replicability, particularly regarding the ratio of host-to-contaminant DNA expected in the raw reads.   2. Lack of Repeat Analysis While the report provides a count of protein-coding genes (16,242), it lacks a summary of repetitive elements. For a genome of nearly 1 Gb (~967 Mb), which is relatively large for a lepidopteran, the proportion of transposable elements (TEs) and simple repeats is a critical descriptive metric. A simple RepeatMasker summary table would significantly enhance the characterization of the genome without requiring deep evolutionary analysis. 3. Comparative Context The manuscript reports the genome size (966.90 Mb) and gene count without context. A brief sentence comparing this size to other sequenced Erebidae or Lymantria species would help orient the reader. Is this genome expanded? Is the gene count typical?    4. Haplotype Divergence The assembly represents one haplotype, with the alternate deposited separately. The authors removed 6 haplotypic duplications. Given that L. dispar can be invasive and genetically diverse, a brief metric describing the heterozygosity rate observed between the two haplotypes would be a valuable addition for researchers planning population genomics studies based on this reference. This manuscript presents a genome assembly of remarkably high quality with comprehensive quality control metrics (BlobToolKit, BUSCO, Merqury). The comments listed above represent opportunities for minor clarifications that would improve the utility of the report, but do not undermine the validity of the dataset or the technical rigor of the work.

Are sufficient details of methods and materials provided to allow replication by others?

Yes

Is the rationale for creating the dataset(s) clearly described?

Yes

Are the datasets clearly presented in a useable and accessible format?

Yes

Are the protocols appropriate and is the work technically sound?

Yes

Reviewer Expertise:

Arthropod Genomics

I confirm that I have read this submission and believe that I have an appropriate level of expertise to confirm that it is of an acceptable scientific standard.

# Reviewer report: Reviewer response for version 1

**DOI:** 10.21956/wellcomeopenres.26066.r139062

**Published:** 2025-12-26

**Authors:** Ricardo Rodriguez-de-la-vega

**Affiliations:** 1 Université Paris-Saclay, Gif-sur-Yvette, France

Comments on Gardiner and ToL "The genome sequence of the gypsy moth..."

 Disclaimer: I received the invitation to post-publication reviewing very late, two approving reviews have already been provided (from April and late November).

 The genome assembly is based on HiFi reads scaffolded with Arima Hi-C data. As is common for assemblies released via the Tree of Life gateway, the quality metrics are excellent: 99.94% of the assembly is anchored into 32 chromosome-level scaffolds, k-mer completeness for the combined primary and alternate haplotypes is 99.35%, and BUSCO scores are near perfect. When first made available in January 2025, this represented approximately the 70th Lepidoptera genome with assembled Z and W sex chromosomes. There is little to add regarding the technical methods or the highly standardized presentation typical of ToL Data Notes.

 Nevertheless, I would like to highlight three issues I would like to see addressed in an eventual revised version.

 1. The common name used in the title has been deprecated by at least one major entomological society. I encourage the authors to change the title to "The genome sequence of the spongy moth..." to align with current recommended nomenclature and avoid derogatory terms.

 2. The RNA-seq data that would have been used for gene annotation is not declared in the Data Note. It is also not listed in the corresponding Tree of Life quality control resource (https://tolqc.cog.sanger.ac.uk/darwin/insects/Lymantria_dispar/). This information should be added for transparency and reproducibility.

 3. A chromosome-level assembly for the same species was published in 2023 (Xu et al., doi: 10.1038/s41597-023-02823-7). This Data Note should include at least a reference to this prior work for proper context.

 4. As observed in many other ToL Data Notes for Lepidoptera genomes, a few large scaffolds contains all sequence with no phylum assigned. In this case, one scaffold is also an outlier in GC content and corresponds to the W chromosome. This consistency across assemblies (at least among ToL's) is worth noting.

 I am linking the Genomes on a Tree webpage of the corresponding assembly, as it provides an entry point to a large collection of related resources.

 https://goat.genomehubs.org/record?recordId=GCA_963576585.1&result=assembly&taxonomy=ncbi#GCA_963576585.1

Are sufficient details of methods and materials provided to allow replication by others?

Yes

Is the rationale for creating the dataset(s) clearly described?

Yes

Are the datasets clearly presented in a useable and accessible format?

Partly

Are the protocols appropriate and is the work technically sound?

Yes

Reviewer Expertise:

Genomics, Bioinformatics, Evolution

I confirm that I have read this submission and believe that I have an appropriate level of expertise to confirm that it is of an acceptable scientific standard, however I have significant reservations, as outlined above.

## References

[1] : Chromosome-level genome assembly of the Asian spongy moths Lymantria dispar asiatica. *Scientific Data* .2023;10(1) : 10.1038/s41597-023-02823-7 10.1038/s41597-023-02823-7 38092795 PMC10719281

[2] : Genomes on a Tree (GoaT): A versatile, scalable search engine for genomic and sequencing project metadata across the eukaryotic tree of life. *Wellcome Open Research* .2023;8: 10.12688/wellcomeopenres.18658.1 10.12688/wellcomeopenres.18658.1 PMC997166036864925

# Reviewer report: Reviewer response for version 1

**DOI:** 10.21956/wellcomeopenres.26066.r139060

**Published:** 2025-11-27

**Authors:** Maurijn van der Zee

**Affiliations:** 1 Leiden University, Leiden, South Holland, The Netherlands

Although some citations in the introduction are not very tractable, this data note is very good. The presented genome of the gypsy moth is of high quality, particularly because very complete chromosome-level scaffolds could be made using Hi-C data and manual curation. The BUSCO score of 98.9% confirms this completeness. Finally, it should be noted that gene annotation has been provided as well in Ensembl Rapid Release in EBI (European Bioinformatics Institute) using Ensembl Gemnebuild and transcriptomic data. This genome will be very useful to the scientific community.

Are sufficient details of methods and materials provided to allow replication by others?

Yes

Is the rationale for creating the dataset(s) clearly described?

Yes

Are the datasets clearly presented in a useable and accessible format?

Yes

Are the protocols appropriate and is the work technically sound?

Yes

Reviewer Expertise:

Evo-devo, insects, comparative genomics

I confirm that I have read this submission and believe that I have an appropriate level of expertise to confirm that it is of an acceptable scientific standard.

# Reviewer report: Reviewer response for version 1

**DOI:** 10.21956/wellcomeopenres.26066.r118046

**Published:** 2025-04-01

**Authors:** Frederique Hilliou

**Affiliations:** 1 Institut Sophia Agrobiotech, Universite Cote d'Azur, INRAE, Nice, Provence-Alpes-Côte d'Azur, France

The manuscript describes the obtention of chromosome level genome (one of the two haplotype was recovered) for Gypsy moth ( *Lymantria dispar*). Sexual chromosomes were identified as the Z (45.03Mb, the largest chromosome) and W (7.87 Mb). A bias in GC % content was found for the W chromosome with 44% GC while all the other chromosomes have a GC content around 38-40%. The mitochondrial genome was also assembled. 16242 proteins-coding genes were annotated with Ensembl together with 7332 non-coding genes and 42146 gene transcripts. This dataset will participate in a better understanding of the place of Noctuoidea in arthropod phylogeny.

 Is genome annotation using Ensembl the best option for arthropod genomes?

 Do you think of the use another annotation such as the one proposed by Hease-Hill, Crouch and Otto will give better results? (Annotation and visualization of parasite, fungi and arthropod genomes with Companion; W. Haese-Hill, K. Crouch and T. D. Otto; Nucleic Acids Research 2024 Vol. 52 Issue W1 Pages W39-W44; DOI: 10.1093/nar/gkae378; https://doi.org/10.1093/nar/gkae378)

 Could you comment on the size of this genome noctuidae (966.9 Megabases)? Is it quite big compare to others? Is there many repeats in the genome?

 The authors wrote that tissue from the abdomen was homogenized using a powerMasher II tissue disruptor. I am not sure I understand how the abdomen is isolated from a larval specimen. Please correct the manuscript. In the HiC sample preparation paragraphe , “tissue from abdomen “ is also used. Please correct the text.

Are sufficient details of methods and materials provided to allow replication by others?

Yes

Is the rationale for creating the dataset(s) clearly described?

Yes

Are the datasets clearly presented in a useable and accessible format?

Yes

Are the protocols appropriate and is the work technically sound?

Partly

Reviewer Expertise:

lepidopteran genomes, detoxifiction gene families

I confirm that I have read this submission and believe that I have an appropriate level of expertise to confirm that it is of an acceptable scientific standard.

## References

[1] : Annotation and visualization of parasite, fungi and arthropod genomes with Companion. *Nucleic Acids Res* .2024;52(W1) : 10.1093/nar/gkae378 W39-W44 10.1093/nar/gkae378 38752499 PMC11223846
